# Comparison and Evaluation of the Molecular Typing Methods for Toxigenic *Vibrio cholerae* in Southwest China

**DOI:** 10.3389/fmicb.2018.00905

**Published:** 2018-05-08

**Authors:** Feng Liao, Zhishuo Mo, Meiling Chen, Bo Pang, Xiaoqing Fu, Wen Xu, Huaiqi Jing, Biao Kan, Wenpeng Gu

**Affiliations:** ^1^Department of Respiratory Medicine, First People’s Hospital of Yunnan Province, Kunming, China; ^2^The Third Affiliated Hospital of Sun Yat-sen University, Guangzhou, China; ^3^National Institute for Communicable Disease Control and Prevention, Chinese Center for Disease Control and Prevention, State Key Laboratory of Infectious Disease Prevention and Control, Collaborative Innovation Center for Diagnosis and Treatment of Infectious Diseases, Beijing, China; ^4^Department of Acute Infectious Diseases Control and Prevention, Yunnan Provincial Centre for Disease Control and Prevention, Kunming, China; ^5^Institute of Medical Biology, Chinese Academy of Medical Science and Peking Union Medical School, Kunming, China

**Keywords:** *Vibrio cholerae*, molecular typing methods, pulsed field gel electrophoresis, multilocus sequence typing, southwest China

## Abstract

*Vibrio cholerae* O1 strains taken from the repository of Yunnan province, southwest China, were abundant and special. We selected 70 typical toxigenic *V. cholerae* (69 O1 and one O139 serogroup strains) isolated from Yunnan province, performed the pulsed field gel electrophoresis (PFGE), multilocus sequence typing (MLST), and MLST of virulence gene (V-MLST) methods, and evaluated the resolution abilities for typing methods. The *ctxB* subunit sequence analysis for all strains have shown that cholera between 1986 and 1995 was associated with mixed infections with El Tor and El Tor variants, while infections after 1996 were all caused by El Tor variant strains. Seventy *V. cholerae* obtained 50 PFGE patterns, with a high resolution. The strains could be divided into three groups with predominance of strains isolated during 1980s, 1990s, and 2000s, respectively, showing a good consistency with the epidemiological investigation. We also evaluated two MLST method for *V. cholerae*, one was used seven housekeeping genes (*adk*, *gyrB*, *metE*, *pntA*, *mdh*, *purM*, and *pyrC*), and all the isolates belonged to ST69; another was used nine housekeeping genes (*cat*, *chi*, *dnaE*, *gyrB*, *lap*, *pgm*, *recA*, *rstA*, and *gmd*). A total of seven sequence types (STs) were found by using this method for all the strains; among them, *rstA* gene had five alleles, *recA* and *gmd* have two alleles, and others had only one allele. The virulence gene sequence typing method (*ctxAB*, *tcpA*, and *toxR*) showed that 70 strains were divided into nine STs; among them, *tcpA* gene had six alleles, *toxR* had five alleles, while *ctxAB* was identical for all the strains. The latter two sequences based typing methods also had consistency with epidemiology of the strains. PFGE had a higher resolution ability compared with the sequence based typing method, and MLST used seven housekeeping genes showed the lower resolution power than nine housekeeping genes and virulence genes methods. These two sequence typing methods could distinguish some epidemiological special strains in local area.

## Introduction

*Vibrio cholerae* is a Gram-negative intestinal pathogen, causing serious human diarrhea, mainly distributed in southern Asia, parts of Africa, Latin America, and other regions (Heidelberg et al., 2000; Morris, 2011). Toxigenic *V. cholerae* is the strain carrying cholera toxin (CT), and mainly refers to O1 and O139 serogroup (Faruque et al., 1998; Nair et al., 2006). However, non-O1/non-O139 *V. cholerae* is not carrying CT, and can only cause mild diarrhea diseases (Singh et al., 2001). Therefore, the prevention and control of toxic strains are more important for humans. In China, cholera was considered to be one of the most serious infectious diseases, although the incidence rate has been maintained at a relatively low level in recent years, the epidemic or outbreak still existed in few areas (Gu et al., 2014). It is very important to perform the molecular typing research for toxigenic *V. cholerae* and clarify the variation and changes of bacteria.

At present, the majority molecular typing methods of *V. cholerae* comprised of pulsed field gel electrophoresis (PFGE), multilocus sequence typing (MLST), MLVA (multiple-locus variable number tandem repeat analysis), or genome sequencing (Karaolis et al., 2001; O’Shea et al., 2004a,b; Danin-Poleg et al., 2007; Grim et al., 2010; Taviani et al., 2010; Okada et al., 2012; Sealfon et al., 2012; Tran et al., 2012). PFGE is considered to have highly discrimination efficiency, and commonly used in the epidemiological or outbreak investigation. Two MLST typing methods have been reported, one was used seven housekeeping genes for *adk*, *gyrB*, *metE*, *pntA*, *mdh*, *purM*, and *pyrC*, established by Octavia^1^ (Octavia et al., 2013). This method has established the database, and researchers in different countries could submit and compare their results. Another was used nine housekeeping genes for *cat*, *chi*, *dnaE*, *gyrB*, *lap*, *pgm*, *recA*, *rstA*, and *gmd*. This method was developed by Garg et al. (2003), several studies have used this method to perform their researches, showing a good discriminatory power (Bhattacharya et al., 2006; Ang et al., 2010). However, this method has not yet established a public database. Researchers from different regions were unable to exchange and share their data. In addition, some studies performed the molecular typing researches by using virulence genes; the results also had effective resolving abilities (Rivera et al., 2001). Up to present, there was no systemic evaluation for molecular typing methods of toxigenic *V. cholerae*, especially for two MLST methods mentioned above. The applicability of different typing methods was still unknown.

Yunnan located in southwest China, bordering Myanmar, Vietnam, and Laos, has an extended frontier. *V. cholerae* resources here were abundant and special, indicated that the cholera was endemic in these regions. Although cholera cases were seldom found in recent years, the imported strains from neighboring countries still existed (Liao et al., 2016). It was very important to find the epidemic consistency of cholera by molecular typing methods. In this study, we selected 70 typical toxigenic *V. cholerae* isolated from different areas and years in Yunnan province, performed the PFGE, two MLST typing, and MLST of virulence gene (V-MLST) methods, and compared the distinguish ability for different molecular typing methods in local epidemic area.

## Materials and Methods

### Strains

Seventy *V. cholerae* strains (already-existing collections) were isolated from different regions, years, and sources in Yunnan province between 1986 and 2012. Sixty-nine strains were O1 serogroup, included 43 Ogawa and 26 Inaba serotype, and one O139 serogroup isolates (we only have three O139 serogroup strains, and selected one as the representative for the study purpose). Fifty-four strains were isolated from the feces samples of patients, 11 from water samples, and five from the external environment (surface of objects), as shown in **Table 1**.

**Table 1:** The 70 *V. cholerae* strains used in this study.

Year	County	Serotype			Source			*rstR*	*tcpA*	*ctxB* subunit	
						
		Ogawa	Inaba	O139	Patient	Water	Environment			Classical	El Tor
1986	Gengma	–	14	–	11	3	–	ET(10)/ET,CL(4)	ET	7	7
1989	Gengma	2	1	–	2	1	–	ET	ET	1	2
	Ruili	2	1	–	3	–	–	ET(2)/ET,CL(1)	ET	2	1
1991	Gengma	1	2	–	3	–	–	ET(2)/ET,CL(1)	ET	1	2
	Ruili	1	–	–	1	–	–	ET,CL	ET	–	1
1994	Yuanmou	–	2	–	2	–	–	ET(1)/ET,CL(1)	ET	2	–
1995	Gengma	3	1	–	3	–	1	ET(1)ET,CL(3)	ET	1	3
	Ruili	2	–	–	2	–	–	ET,CL	ET	1	1
	Jinghong	2	1	–	2	1	–	ET,CL	ET	1	2
	Longchuan	1	–	–	1	–	–	ET,CL	ET	1	–
	Mangshi	1	–	–	1	–	–	ET,CL	ET	–	1
	Dali	1	–	–	1	–	–	ET,CL	ET	1	–
1996	Yongshan	2	1	–	–	3	–	ET,CL	ET	3	–
1997	Yuanmou	–	1	–	1	–	–	ET,CL	ET	1	–
	Wuding	–	1	–	1	–	–	ET,CL	ET	1	–
1998	Ruili	6	–	1	4	–	3	ET(1)/ET,CL(6)	ET	6	1
	Yanshan	2	–	–	2	–	–	ET,CL	ET	2	–
	Guangnan	2	–	–	2	–	–	ET,CL	ET	2	–
	Mangshi	3	–	–	2	–	1	ET,CL	ET	3	–
1999	Gejiu	1	–	–	1	–	–	ET,CL	ET	1	–
	Kunming	1	–	–	1	–	–	ET,CL	ET	1	–
	Yuanyang	3	–	–	1	2	–	ET,CL	ET	3	–
	Dali	1	–	–	–	1	–	ET,CL	ET	1	–
2001	Mangshi	3	1	–	4	–	–	ET,CL	ET	4	–
2011	Ruili	2	–	–	2	–	–	ET,CL	ET	2	–
2012	Ruili	1	–	–	1	–	–	ET,CL	ET	1	–


*ET, El Tor biotype; CL, Classical biotype. The numbers in the bracket represented the strains possessed the ET or ET, CL alleles in different counties and years, no bracket represented all the isolates had the same allele.*

### PCR Detection of Virulence Genes and *ctxB* Sequencing

Genomic DNA was extracted from each isolate using a DNA extraction kit (Tiangen, Beijing) according to the manufacturers’ instructions. The virulence genes for *ctxAB*, *ompU*, *ace*, *zot*, *toxR*, *rtxC*, and CTX phage *rstR* (Classical/El Tor) and *tcpA* (Classical/El Tor) were amplified using Taq premix (TaKaRa, Japan), the primers and amplification procedures were as described previously (Chow et al., 2001; Singh et al., 2001, 2002; O’Shea et al., 2004b). All of the strains were sequenced for *ctxB* gene subunit to further identify the characters of the CTX phage, Taq premix (TaKaRa, Japan) was used as described above, and amplification processes were performed as previously described (Goel et al., 2010). The amplification products were sent for bidirectional sequencing (TaKaRa, Japan), and the results were analyzed using DNAStar (DNASTAR, Inc., United States) and MEGA 4 software (Tamura et al., 2007). The *ctxB* sequences of N16961 of El Tor *V. cholerae* (GenBank: NC-002505) and O395 Classical strain (GenBank: NC-012582) were used as the standards for comparison.

### Pulsed Field Gel Electrophoresis

Pulsed field gel electrophoresis was performed based on the PulseNet protocol for *V. cholerae* and procedures described previously (Gu et al., 2014). The enzyme digestion for each plug was *Not*I 40U at 37°C for 4 h. The CHEF-Mapper (Bio-Rad) was used for electrophoresis, and the pulse time ranged from 1 to 20 s for 13 h, and 20 to 25 s for 6 h. The gel was stained using Gel Red (Biotium) and visualized using the gel imaging system (Bio-Rad, Gel Doc XR). PFGE patterns were analyzed with BioNumerics version 6.6 (Applied Maths, Belgium), and a dendrogram was produced using the Dice coefficient and un-weighted pair group method with arithmetic mean algorithm (UPGMA). A pairwise distance matrix was also created.

### MLST and V-MLST

#### Seven Housekeeping Genes

PCR amplification was performed according to the public database (see text footnote 1) for *adk*, *gyrB*, *metE*, *pntA*, *mdh*, *purM*, and *pyrC*, and a list of primers were shown in **Table 2**. A 100 μl reaction system was used, including 50 μl Taq premix (TaKaRa, Japan), 40 μl water, upstream and downstream of primers 2.5 μl, respectively, and template 5 μl. Amplification procedure was: 94°C 5 min; 94°C 15 s, 50°C 30 s, 72°C 30 s, 35 cycles; the last 72°C 10 min. Amplified products were sent to bidirectional sequencing (TaKaRa, Japan).

**Table 2:** The primers used in this study.

Group	Gene	Gene product	Sequence (5′–3′)		Length (bp)	Reference
					
			Forward	Reverse		
Seven genes	*adk*	Adenylate kinase	CATCATTCTTCTCGGTGCTC	AGTGCCGTCAAACTTCAGGTA	416	Octavia et al., 2013
	*gyrB*	DNA gyrase subunit B	GTACGTTTCTGGCCTAGTGC	GGGTCTTTTTCCTGACAATC	431	Octavia et al., 2013
	*metE*	Methionine synthase	CGGGTGACTTTGCTTGGT	CAGATCGACTGGGCTGTG	421	Octavia et al., 2013
	*mdh*	Malate dehydrogenase	ATGAAAGTCGCTGTTATTGG	GCCGCTTGGCCCATAGAAAG	591	Octavia et al., 2013
	*pntA*	Pyridine nucleotide transhydrogenase	CTTTGATGGAAAAACTCTCA	GATATTGCCGTCTTTTTCTT	431	Octavia et al., 2013
	*purM*	Phosphoribosyl-formylglycinamide cyclo-ligase	GGTGTCGATATTGATGCAGG	GGAATGTTTTCCCAGAAGCC	476	Octavia et al., 2013
	*pyrC*	Dihydroorotase	ATCATGCCTAACACGGTTCC	TTCAAACACTTCGGCATA	449	Octavia et al., 2013
Nine genes	*cat*	Catalase-peroxidase	ATGGCTTATGAATCGATGGG	TCCCATTGCCATGCACC	543	Garg et al., 2003
	*chi*	Chitinase	CAYGAYCCRTGGGCWGC	ACRTCTTCAATCTTGTC	366	Garg et al., 2003
	*dnaE*	DNA polymerase III subunit alpha	CGRATMACCGCTTTCGCCG	GAKATGTGTGAGCTGTTTGC	530	Garg et al., 2003
	*gyrB*	DNA gyrase subunit B	GAAGGBGGTATTCAAGC	GAGTCACCCTCCACWATGTA	528	Garg et al., 2003
	*lap*	Aminopeptidase	GAAGAGGTCGGTTTGCGAGG	GTTTGAATGGTGAGCGGTTTGCT	468	Garg et al., 2003
	*pgm*	Phosphoglucomutase	CCKTCSCAYAACCCGCC	TCRACRAACCATTTGAADCC	395	Garg et al., 2003
	*recA*	Recombinase RecA	GAAACCATTTCGACCGGTTC	CCGTTATAGCTGTACCAAGCGCCC	744	Garg et al., 2003
	*rstA*	RstA phage-related replication protein	CGTGTTAGAGCACAC	GAGTGAATCGTCGTG	539	Garg et al., 2003
	*gmd*	GDP-mannose 4,6-dehydratase	CTAGAAGCCCTTATGCTGTG	GTAATTTCTGGCACCCATCC	481	This study
Virulence genes	*ctxAB*	Cholera toxin subunit A and B	ATGCCGCGCCACATAATACG	AAGCGCTGTGGGTAGAAGTG	691	This study
	*tcpA*	Toxin coregulated pilin A	GGTGGGCATAGTGATAAGAG	CGCCTCCAATAATCCGACAC	1050	This study
	*toxR*	Transcriptional regulator R	AATACCCATGGCGATGTGTC	GGGAGATACTGGGACATTAG	827	This study


#### Nine Housekeeping Genes

PCR amplification was made following the published work (Garg et al., 2003) targeting the genes *cat*, *chi*, *dnaE*, *gyrB*, *lap*, *pgm*, *recA*, *rstA*, and *gmd*. The primers were shown in **Table 2**, *gmd* gene could not amplified by reference primer, so we designed the new primers by using Clone Manager Professional 8.0 software (Scientific & Educational), and the *gmd* gene of *V. cholerae* reference strain N16961 was used. The reaction system was identical as mentioned above. Amplification procedure was: 94°C 5 min; 94°C 15 s, 55°C 30 s, 72°C 30 s, 35 cycles; the last 72°C 10 min. Amplified products were sent to bidirectional sequencing (TaKaRa, Japan).

#### Virulent Genes of MLST

We designed the *ctxAB*, *tcpA*, and *toxR* genes primers (**Table 2**) by using Clone Manager Professional 8.0 software (Scientific & Educational) as well, *V. cholerae* reference strain N16961 was also used. The reaction system and amplification procedure were identical as mentioned above. Amplified products were sent to bidirectional sequencing (TaKaRa, Japan).

### Data Analysis

All the sequencing results were assembled by DNAStar 6.0 software (DNASTAR, Inc., United States), compared and aligned by MEGA 4.0 (Tamura et al., 2007). The seven housekeeping genes sequences were submitted to the public database (see text footnote 1), the alleles of different genes and sequence types (STs) were obtained. The sequence alignments were performed for nine housekeeping and virulence genes, when a new sequence appeared; we gave a new allele for each gene, and finally got the STs by permutation and combination of the nine genes or virulent genes. The minimum spanning tree was constructed by using BioNumerics 6.6 software (Applied Maths, Belgium) for sequences based typing methods.

### Nucleotide Sequence Accession Numbers

All the genes of different sequences were deposited in the GenBank with the accession numbers: KX960341 to KX960367.

### Ethics Approval Statement

The human sample collection and detection protocols were carried out in accordance with relevant guidelines and regulations approved by Ethical Committee of Yunnan Provincial Centre for Disease Control and Prevention. All experimental procedures were approved by the Ethics Review Committee [Institutional Review Board (IRB)] of Yunnan Provincial Centre for Disease Control and Prevention. All adult subjects provided informed consent, and a parent or guardian of any child participant provided informed consent on their behalf. The informed consents were oral for all the participants, because the samples were too large; we could not get all the written ones. All samples collections and experimental procedures were approved by the Ethics Review Committee, according to Chinese ethics laws and regulations. The anonymization strategy was used for the human sample collection and detection protocols used in this study. The details of patients, such as name, address, age, and sex were anonymous, and we just defined the numbers of patients or samples.

## Results

### PCR Test for Virulence Genes and *ctxB* Sequencing

The *ctxAB*, *ompU*, *ace*, *zot*, *toxR*, and *rtxC* for all of the isolates were positive; *tcpA*^ElTor^ was positive for all of the isolates as well, while *tcpA*^Classical^ was negative. For the *rstR*, most of the strains carried *rstR*^ElTor^ and *rstR*^Classical^; however, some of the strains possessed only *rstR*^ElTor^. The *ctxB* subunit showed mixed infection with El Tor type and El Tor variant strains before 1995; after 1996 all of the isolates harbored the *ctxB* Classical except one O139 *V. cholerae* that possessed *ctxB* El Tor (**Table 1**).

### PFGE Results

**FIGURE 1:**
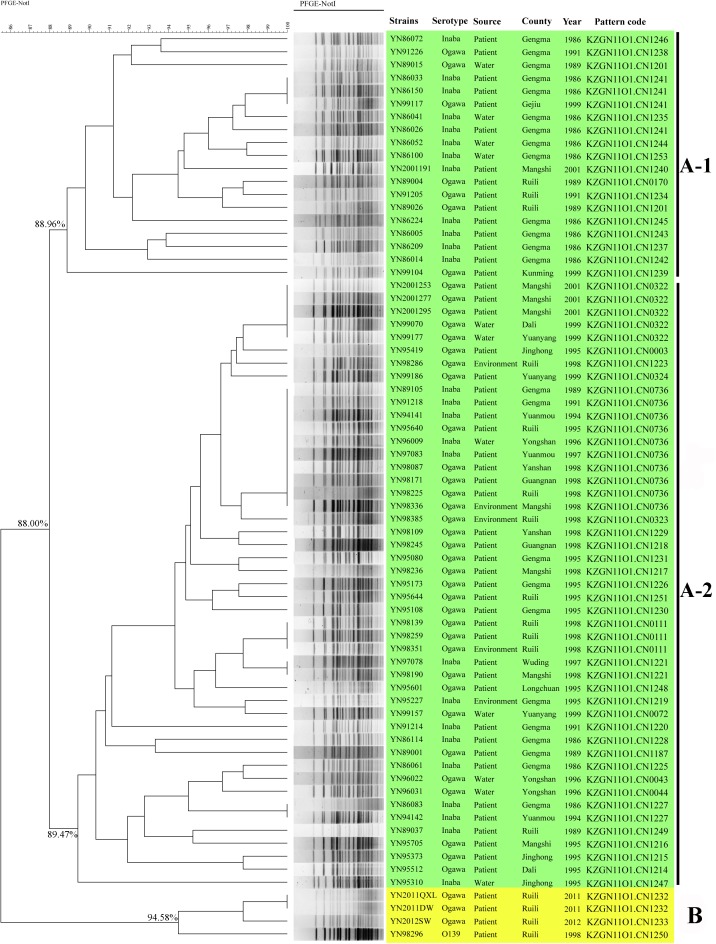
PFGE-*Not*I dendrogram for 70 *V. cholerae* in this study. The green areas of group A mainly referred to native epidemic strains, while the yellow areas of group B were mostly imported strains from Myanmar.

Seventy toxigenic *V. cholerae* obtained 50 PFGE patterns, with a high resolution. The clusters could be divided into three groups, named as A-1, A-2, and B with predominance of strains isolated during 1980s, 1990s, and 2000s, respectively (**Figure 1**). A total of 88.00% similarity of PFGE pattern was found between all the isolates and the pattern similarity scale was 88.96% for group A-1, 89.47% for group A-2, and 94.58% for group B. The green areas of group A-1 and A-2 mainly referred to native epidemic strains, while the yellow areas of group B were mostly imported strains from Myanmar for epidemiological investigation, except one O139 strain. Some *V. cholerae* isolated in different years and areas had identical PFGE patterns, such as Gengma in 1986 and Gejiu in 1999 (KZGN11O1.CN1241); Mangshi in 2001, Dali and Yuanyang in 1999 (KZGN11O1.CN0322); and Gengma in 1989 and 1991, Yuanmou in 1994 and 1997, Ruili in 1995 and 1998, Yongshan in 1996, Yanshan, Guangnan, and Mangshi in 1998 (KZGN11O1.CN0736). Compared the PFGE result with our previous study (Liao et al., 2016), we found that PFGE had highly discrimination power with whole genomic sequencing method, since the imported strain YN2011QXL (YN2011004) was separated from other three *V. cholerae* in our previous work used genomic sequencing. And in this study, YN2011QXL was also clustered to different groups with other *V. cholerae*.

### MLST and V-MLST Results

**FIGURE 2:**
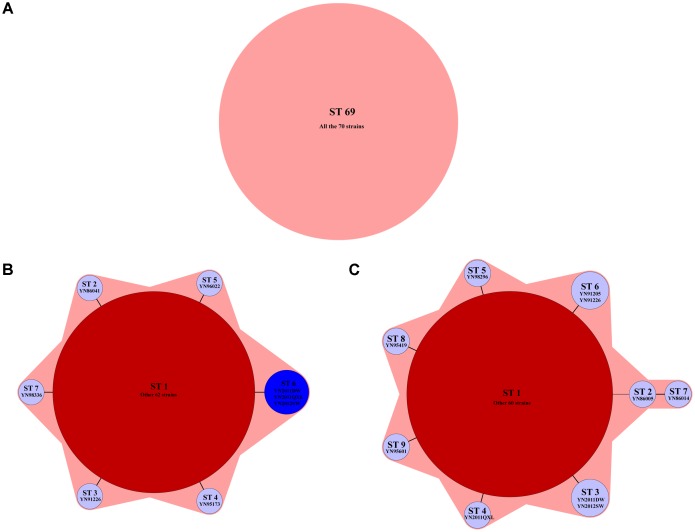
The minimum spanning tree of three sequence based genotyping methods. **(A)** Seven housekeeping genes: all the strains belonged to ST69; **(B)** nine housekeeping genes: seven STs were found by using this method, named as ST1–ST7. The typical isolates were shown below each STs; and **(C)** virulence genes: nine STs were found by using this method, named as ST1–ST9. The typical isolates were shown below each STs.

The MLST results used seven housekeeping genes showed that all the strains belonged to ST69. *adk* allele was 7, *gyrB* was 11, *metE* 37, *pntA* 12, *mdh* 4, *purM* 1, and *pyrC* 20. The results had no relations with isolated areas or years of the strains (**Figure 2A**). Nine housekeeping genes were arranged and combined to produce seven different STs, named as ST1–ST7, as shown in **Figure 2B**. Three imported strains from Myanmar after 2011 formed their own ST (**Figure 2B**, blue area). Three virulence genes were analyzed and produced nine STs, named as ST1–ST9, as shown in **Figure 2C**. The imported strains also formed their own STs, while YN2011QXL and other two strains were divided into different types. The latter two sequences based typing methods had consistency with epidemiology of the strains.

**FIGURE 3:**
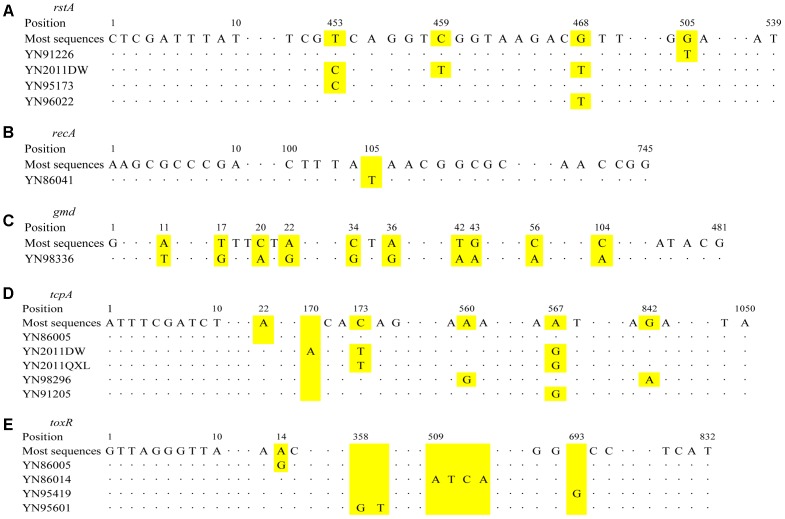
Schematic diagram of base position changes for different genes. **(A)** *rstA*; **(B)** *recA*; **(C)** *gmd*; **(D)** *tcpA*; and **(E)** *toxR*. The yellow areas for each gene represent the changes of base positions.

Seven STs were found for all the isolates used nine housekeeping genes method, *rstA* gene had five alleles (YN2011DW, YN2011QXL, and YN2012SW; YN91226; YN95173; YN96022; and other strains), *recA* had two alleles (YN86041 and other strains), *gmd* had two alleles (YN98336 and other strains). Other six housekeeping genes had only one allele, respectively. For *rstA* gene, YN91226 mutated at position 505 nt; YN2011DW mutated at 453, 459, and 468 nt; YN95173 mutated at 453 nt; and YN96022 mutated at 468 nt, as **Figure 3A** shown. For *recA* gene, YN86041 inserted a “T” at position 105 nt (**Figure 3B**). For *gmd* gene, YN98336 mutated at 11, 17, 20, 22, 34, 36, 42, 43, 56, and 104 nt position (**Figure 3C**).

The virulence genes sequences results showed *ctxAB* gene was identical for all the strains, *tcpA* gene had six alleles (YN86005 and YN86014; YN2012SW and YN2011DW; YN2011QXL; YN98296; YN91205 and YN91226; and other strains), *toxR* gene had five alleles (YN86005; YN95601; YN86014; YN95419; and other strains). For *tcpA*, YN86005 deleted an “A” at position 22 nt; YN2011DW mutated at 173 and 567 nt, and inserted an “A” at 170 nt; YN2011QXL mutated at 173 and 567 nt; YN98296 mutated at 560 and 842 nt; YN91205 mutated at 567 nt, as **Figure 3D** shown. For *toxR* gene, YN86005 mutated at 14 nt; YN86014 inserted “ATCA” at 509 nt; YN95419 inserted a “G” at 693 nt; and YN95601 inserted “GT” at 358 nt (**Figure 3E**).

All the sequence alignments results for genotyping methods were shown in Supplementary Material.

### Comparison the Molecular Typing Methods

Compared the sequence based typing methods in this study, genotyping of seven housekeeping genes was unable to distinguish between strains from different epidemiological resources; genotyping of nine housekeeping genes divided 70 strains into seven STs, and the different epidemiological resources of isolates were distinguished by this method; genotyping of three virulence genes had the similar discriminatory power with nine housekeeping genes. However, the discriminatory ability based on sequence typing methods was lower than PFGE in the local epidemic areas. For example, the cholera epidemic happened in 1986 of Gengma County (A-1 group); 10 patterns were found among 11 strains, while only two STs were found used nine housekeeping genes method (ST1 and ST2); and three STs were identified used virulence genes method (ST1, ST2, and ST7).

## Discussion

Pulsed field gel electrophoresis is considered to be the “golden standard” for pathogen molecular typing techniques, showing the highly resolution power, frequently used for outbreak investigation and traceability analysis. MLST is often used for analysis the long-term variability and changes of strains. The purpose of our study was evaluated different molecular typing methods for toxigenic *V. cholerae* in Yunnan province. From our previous works (Gu et al., 2014; Liao et al., 2016), *V. cholerae* in Yunnan province, southwest China had similar homology with strains from other part of China, or even some southeast Asia countries. Therefore, the isolates used in this study could reflect the general *V. cholerae* distributions characteristics in China or southeast Asia, and have enough representatives of the bacteria.

At present, PFGE have the standard experimental procedure, the data could be exchanged and analyzed between different laboratories. Two MLST methods have been reported, Garg et al. (2003) analyzed 96 O139 strains used nine housekeeping genes in 2003, and they found 64 new alleles in 51 STs. Several studies have used this method to perform their researches, for example, Nguyen et al. (2009) performed the MLST method for cholera outbreak in Vietnam in 2009 by using nine housekeeping genes, and all the strains had the same ST with N16961. Lee et al. (2006) analyzed the Mozambique *V. cholerae* by using nine genetic loci showed that the Mozambique isolates have the same ST as O1 El Tor N16961. Kotetishvili et al. (2003) used 22 *V. cholerae* isolates to perform the PFGE and MLST by using three housekeeping genes, *gyrB*, *pgm*, and *recA*; sequence data were also obtained for the virulence-associated genes *tcpA*, *ctxA*, and *ctxB*. Their results showed that MLST had better discriminatory ability than PFGE; On MLST analysis, there was clear clustering of epidemic serogroups; much greater diversity was seen among *tcpA* and *ctxAB* positive *V. cholerae* strains from others, non-epidemic serogroups, with a number of *tcpA* and *ctxAB* alleles identified. However, this method has not established public database, and its application was limited. Octavia et al. (2013) developed a new MLST method for *V. cholerae* in 2013; they found a total of 77 isolates were divided into 66 STs, including 55 non-O1/non-O139 strains. While, in this study, 70 toxigenic strains had only one ST, no correction with epidemiological information could be found with the typing results, we considered this method was more suitable for genomic diversity of non-O1/non-O139 *V. cholerae*. In fact, in our study, PFGE had higher discriminatory power than all sequence based typing method. The cholera epidemic happened in 1986 of Gengma County (A-1 group); 10 patterns were found among 11 strains, while only two STs were found used nine housekeeping genes method (ST1 and ST2); and three STs were identified used virulence genes method (ST1, ST2, and ST7). Therefore, we considered that PFGE was more suitable for molecular typing in cholera epidemic local area.

In Boyd and Waldor (2002) study, genetic variation at the *tcpA* locus in toxigenic isolates of *V. cholerae* was investigated; the results showed *tcpA* sequences were far more diverse than other loci. This diversity was a reflection of diversifying selection in adaptation to the host immune response. Therefore, we selected three major virulence genes of *V. cholerae* to perform the molecular typing analysis. Its discriminatory ability was similar with nine housekeeping genes method, and the *tcpA* gene discriminatory effect was the best compared with *ctxAB* and *toxR*.

Compared the sequence based typing methods in this study, genotyping of seven housekeeping genes was unable to distinguish between strains from different epidemiological resources; genotyping of nine housekeeping genes divided 70 strains into seven STs, and the different epidemiological resources of isolates were distinguished by this method; genotyping of three virulence genes had the similar discriminatory power with nine housekeeping genes. However, the discriminatory ability based on sequence typing methods was lower than PFGE in the local epidemic areas.

## Author Contributions

BK, HJ, and WG designed the work. FL, MC, BP, XF, and WX did the experiments. ZM and WG analyzed the data. ZM drafted the work. BK and HJ revised it critically for important intellectual content.

## Conflict of Interest Statement

The authors declare that the research was conducted in the absence of any commercial or financial relationships that could be construed as a potential conflict of interest.

## Footnotes

1 http://pubmlst.org/vcholerae

## References

[B1] Ang G. Y. Yu C. Y. Balqis K. Elina H. T. Azura H. Hani M. H. (2010). Molecular evidence of cholera outbreak caused by a toxigenic *Vibrio cholerae* O1 El tor variant strain in Kelantan, Malaysia. 48 3963–3969. 10.1128/JCM.01086-10 20826646 PMC3020861

[B2] Bhattacharya T. Chatterjee S. Maiti D. Bhadra R. K. Takeda Y. Nair G. B. (2006). Molecular analysis of the rstR and orfU genes of the CTX prophages integrated in the small chromosomes of environmental *Vibrio cholerae* non-O1, non-O139 strains. 8 526–634. 10.1111/j.1462-2920.2005.00932.x 16478458

[B3] Boyd E. F. Waldor M. K. (2002). Evolutionary and functional analyses of variants of the toxin-coregulated pilus protein TcpA from toxigenic *Vibrio cholerae* non-O1/non-O139 serogroup isolates. 148 1655–1666. 10.1099/00221287-148-6-1655 12055286

[B4] Chow K. H. Ng T. K. Yuen K. Y. Yam W. C. (2001). Detection of RTX toxin gene in *Vibrio cholerae* by PCR. 39 2594–2597. 10.1128/JCM.39.7.2594-2597.2001 11427575 PMC88191

[B5] Danin-Poleg Y. Cohen L. A. Gancz H. Broza Y. Y. Goldshmidt H. Malul E. (2007). *Vibrio cholerae* strain typing and phylogeny study based on simple sequence repeats. 45 736–746. 10.1128/JCM.01895-06 17182751 PMC1829105

[B6] Faruque S. M. Albert M. J. Mekalanos J. J. (1998). Epidemiology, genetics, and ecology of toxigenic *Vibrio cholerae*. 62 1301–1314. 10.1128/mmbr.62.4.1301-1314.1998 PMC98947 9841673

[B7] Garg P. Aydanian A. Smith D. J. Glenn M. J. Nair G. B. Stine O. C. (2003). Molecular epidemiology of O139 *Vibrio cholerae*: mutation, lateral gene transfer, and founder flush. 9 810–814. 10.3201/eid0907.020760 12890320 PMC3023423

[B8] Goel A. K. Jain M. Kumar P. Jiang S. C. (2010). Molecular characterization of *Vibrio cholerae* outbreak strains with altered El Tor biotype from southern India. 26 281–287. 10.1007/s11274-009-0171-7 20495624 PMC2872801

[B9] Grim C. J. Hasan N. A. Taviani E. Haley B. Chun J. Brettin T. S. (2010). Genome sequence of hybrid *Vibrio cholerae* O1 MJ-1236, B-33, and CIRS101 and comparative genomics with *V. cholerae*. 192 3524–3533. 10.1128/JB.00040-10 20348258 PMC2897672

[B10] Gu W. Yin J. Yang J. Li C. Chen Y. Xu W. (2014). Characterization of *Vibrio cholerae* from 1986 to 2012 in Yunnan Province, southwest China bordering Myanmar. 21 1–7. 10.1016/j.meegid.2013.10.015 24177595

[B11] Heidelberg J. F. Eisen J. A. Nelson W. C. Clayton R. A. Gwinn M. L. Dodson R. J. (2000). DNA sequence of both chromosomes of the cholera pathogen *Vibrio cholerae*. 406 477–483. 10.1038/35020000 10952301 PMC8288016

[B12] Karaolis D. K. Lan R. Kaper J. B. Reeves P. R. (2001). Comparison of *Vibrio cholerae* pathogenicity islands in sixth and seventh pandemic strains. 69 1947–1952. 10.1128/IAI.69.3.1947-1952.2001 11179381 PMC98110

[B13] Kotetishvili M. Stine O. C. Chen Y. Kreger A. Sulakvelidze A. Sozhamannan S. (2003). Multilocus sequence typing has better discriminatory ability for typing *Vibrio cholerae* than does pulsed-field gel electrophoresis and provides a measure of phylogenetic relatedness. 41 2191–2196. 10.1128/JCM.41.5.2191-2196.2003 12734277 PMC154734

[B14] Lee J. H. Han K. H. Choi S. Y. Lucas M. E. Mondlane C. Ansaruzzaman M. (2006). Multilocus sequence typing (MLST) analysis of *Vibrio cholerae* O1 El Tor isolates from Mozambique that harbour the classical CTX prophage. 55 165–170. 10.1099/jmm.0.46287-0 16434708

[B15] Liao F. Pang B. Fu X. Xu W. Kan B. Jing H. (2016). The complete genomic analysis of an imported *Vibrio cholerae* from Myanmar in southwest China. 44 272–277. 10.1016/j.meegid.2016.07.023 27448952

[B16] Morris J. G. Jr. (2011). Cholera–modern pandemic disease of ancient lineage. 17 2099–2104. 10.3201/eid1711.111109 22099113 PMC3310593

[B17] Nair G. B. Qadri F. Holmgren J. Svennerholm A. M. Safa A. Bhuiyan N. A. (2006). Cholera due to altered El Tor strains of *Vibrio cholerae* O1 in Bangladesh. 44 4211–4213. 10.1128/JCM.01304-06 16957040 PMC1698305

[B18] Nguyen B. M. Lee J. H. Cuong N. T. Choi S. Y. Hien N. T. Anh D. D. (2009). Cholera outbreaks caused by an altered *Vibrio cholerae* O1 El Tor biotype strain producing classical cholera toxin B in Vietnam in 2007 to 2008. 47 1568–1571. 10.1128/JCM.02040-08 19297603 PMC2681878

[B19] Octavia S. Salim A. Kurniawan J. Lam C. Leung Q. Ahsan S. (2013). Population structure and evolution of non-O1/non-O139 *Vibrio cholerae* by multilocus sequence typing. 8:e65342. 10.1371/journal.pone.0065342 23776471 PMC3679125

[B20] Okada K. Roobthaisong A. Nakagawa I. Hamada S. Chantaroj S. (2012). Genotypic and PFGE/MLVA analyses of *Vibrio cholerae* O1: geographical spread and temporal changes during the 2007-2010 cholera outbreaks in Thailand. 7:e30863. 10.1371/journal.pone.0030863 22292065 PMC3265523

[B21] O’Shea Y. A. Finnan S. Reen F. J. Morrissey J. P. O’gara F. Boyd E. F. (2004a). The Vibrio seventh pandemic island-II is a 26.9 kb genomic island present in Vibrio cholerae El Tor and O139 serogroup isolates that shows homology to a 43.4 kb genomic island in *V. vulnificus*. 150 4053–4063. 15583158 10.1099/mic.0.27172-0

[B22] O’Shea Y. A. Reen F. J. Quirke A. M. Boyd E. F. (2004b). Evolutionary genetic analysis of the emergence of epidemic *Vibrio cholerae* isolates on the basis of comparative nucleotide sequence analysis and multilocus virulence gene profiles. 42 4657–4671. 15472325 10.1128/JCM.42.10.4657-4671.2004 PMC522369

[B23] Rivera I. N. Chun J. Huq A. Sack R. B. Colwell R. R. (2001). Genotypes associated with virulence in environmental isolates of *Vibrio cholerae*. 67 2421–2429. 10.1128/AEM.67.6.2421-2429.2001 11375146 PMC92890

[B24] Sealfon R. Gire S. Ellis C. Calderwood S. Qadri F. Hensley L. (2012). High depth, whole-genome sequencing of cholera isolates from Haiti and the Dominican Republic. 13:468. 10.1186/1471-2164-13-468 22963323 PMC3473251

[B25] Singh D. V. Isac S. R. Colwell R. R. (2002). Development of a Hexaplex PCR assay for rapid detection of virulence and regulatory genes in *Vibrio cholerae* and *Vibrio mimicus*. 40 4321–4324. 10.1128/JCM.40.11.4321-4324.2002 12409420 PMC139685

[B26] Singh D. V. Matte M. H. Matte G. R. Jiang S. Sabeena F. Shukla B. N. (2001). Molecular analysis of *Vibrio cholerae* O1, O139, non-O1, and non-O139 strains: clonal relationships between clinical and environmental isolates. 67 910–921. 10.1128/AEM.67.2.910-921.2001 11157262 PMC92666

[B27] Tamura K. Dudley J. Nei M. Kumar S. (2007). MEGA4: molecular evolutionary genetics analysis (MEGA) software version 4.0. 24 1596–1599. 10.1093/molbev/msm092 17488738

[B28] Taviani E. Grim C. J. Choi J. Chun J. Haley B. Hasan N. A. (2010). Discovery of novel *Vibrio cholerae* VSP-II genomic islands using comparative genomic analysis. 308 130–137. 10.1111/j.1574-6968.2010.02008.x 20528940 PMC2925232

[B29] Tran H. D. Alam M. Trung N. V. Kinh N. V. Nguyen H. H. Pham V. C. (2012). Multi-drug resistant *Vibrio cholerae* O1 variant El Tor isolated in northern Vietnam between 2007 and 2010. 61 431–437. 10.1099/jmm.0.034744-0 22016560 PMC3347965

